# Characteristics of factors for decreased lung function in elderly patients with type 2 diabetes

**DOI:** 10.1038/s41598-019-56759-3

**Published:** 2019-12-27

**Authors:** Masaki Ishii, Yasuhiro Yamaguchi, Hironobu Hamaya, Sumito Ogawa, Mitsuo Imura, Akishita Masahiro

**Affiliations:** 1 The Department of Geriatric Medicine, The University of Tokyo, Tokyo, Japan; 2 Division of Department of Respiratory Medicine, Jichi Medical University Saitama Medical Center, Saitama, Japan; 3 Okamoto Internal Medicine Clinic, Shizuoka, Japan

**Keywords:** Diseases, Medical research, Risk factors

## Abstract

Chronic obstructive pulmonary disease (COPD) often accompanies type 2 diabetes mellitus (T2DM). However, background factors affecting these diseases in the elderly remain unclear. Eligible patients with T2DM were divided into two age groups—non-elderly (<65 years) and elderly (≥65 years); COPD, ratio of forced expiratory volume in one second to forced expiratory volume (FEV1/FVC ratio), and percent predicted forced expiratory volume in one second (FEV1% predicted) were examined, and factors related to reduced respiratory function according to age group were evaluated. In total, 371 patients with T2DM were analysed. COPD was found in 9 patients (5.3%) in the non-elderly group and 45 (22.5%) in the elderly group. In the elderly, male sex, low body mass index (BMI), insulin therapy, and high C-peptide immunoreactivity levels were factors related to COPD. In the non-elderly, age, female sex, high BMI were factors related to decreased FEV1% predicted. Female sex was factor related to decreased FEV1% predicted in both age groups. Low BMI was a factor related to reduced respiratory function in elderly patients and high BMI was a factor related to reduced respiratory function in non-elderly patients. Thus, BMI needs to be managed according to the age and general condition of T2DM patients.

## Introduction

According to figures from 2017, approximately 420 million individuals worldwide are diagnosed with diabetes. Approximately 90% of diabetes patients in high-income countries are type 2 diabetes mellitus (T2DM)^1^. In Japan, it is estimated that more than 10 million people have diabetes, with the rate of increase in incidence particularly high for elderly individuals^2^. Decreased muscle mass and increased visceral fat due to aging increase the risk of developing T2DM through insulin resistance; these factors also increase the risk of geriatric syndromes such as sarcopenia and frailty^3^. Chronic obstructive pulmonary disease (COPD) is a lifestyle-related inflammatory disease of the lungs caused by long-term exposure to harmful substances, such as cigarette smoke. The number of patients with COPD worldwide is at least 210 million^4^ and COPD is the third leading cause of death^5^. In the Nippon COPD Epidemiology (NICE) study in Japan, the prevalence of COPD was estimated to be 8.6% in those over 40 years of age, an approximate total of 5.3 million patients^6^. The prevalence of COPD increases with age, and thus, it is a typical respiratory disease of the elderly.

COPD has many comorbidities including T2DM and these complications exacerbate COPD^7,8^. Inflammation and obesity have been suggested to enable co-existence of T2DM and COPD. Excess adipocytes produce inflammatory cytokines, such as interleukin-6 and tumor necrosis factor-α, which drive systemic inflammation^9^, increasing the risk of development of T2DM^10,11^ and COPD^12^. Obesity reduces the functional residual capacity and expiratory reserve volume through fat accumulation in the abdomen and chest, adversely affecting respiratory function^13,14^. Leone *et al*. examined the relationship between metabolic syndrome and respiratory function in 120,000 men and found that the odds ratios [95% CI] of abdominal obesity to reduced forced expiratory volume in one second (FEV1) and forced vital capacity (FVC) were 1.94 [1.80–2.09] and 2.11 [1.95–2.29], respectively^15^.

Conversely, low body mass index (BMI) has also been reported as a risk factor for COPD. In a group of 105 COPD patients (mean age 64.6 years), Mete *et al*. found 8.6% had a BMI <18 and significantly lower FEV1 and FEV1/FVC ratio^16^. Furthermore, Landbo *et al*. reported that among patients with FEV1/FEV ratio <70%, those with a low BMI had significantly higher mortality compared with those with a normal or high BMI (relative risk [95% CI]: 1.64 [1.20–2.23])^17^. Rates reported for obese patients vary depending on COPD severity ranked according to the Global Initiative for Chronic Obstructive Lung Disease (GOLD): stage 1 (16.1%), stage 2 (23.5%), stage 3 (9.3%), and stage 4 (5.9%)^18^. These data indicate that there are fewer obese patients with severe rather than mild or moderate COPD. COPD progresses with aging, and a patient’s physical activity, vulnerability, and BMI change as COPD becomes more severe. It is plausible that body fat affects both T2DM and respiratory function. However, the relationship between body fat and respiratory function in elderly patients with T2DM remains unclear. Therefore, we examined factors influencing respiratory function in patients of two age groups with T2DM.

## Results

### Patient characteristics

**Figure 1:**
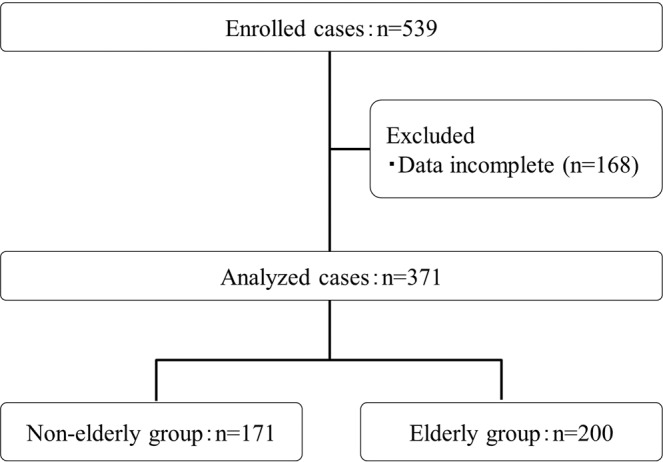
Patient flow. The analysis was carried out in the patients population with data for all items.

Of 539 eligible patients, 168 were excluded from analysis owing to incomplete data (Fig. 1). The remaining 371 patients were divided into either non-elderly (<65 years, n = 171, 46.1%) or elderly (≥65 years, n = 200, 53.9%) groups. Patient characteristics are shown in Table 1. The mean age was 54.3 years for the non-elderly group and 71.3 years for the elderly group; mean BMIs were 27.6 and 24.0, respectively. The rate of patients receiving insulin therapy was 25.1% in the non-elderly group and 70.5% in the elderly group. COPD was found in 5.3% of the non-elderly group and in 22.5% of the elderly group.

**Table 1:** Patient characteristics.

	Non-elderly group (n = 171)	Elderly group (n = 200)
Age (years), mean ± SD	54.3 ± 6.6	71.3 ± 4.6
**Sex, n (%)**		
male	132 (77.2%)	131 (65.5%)
female	39 (22.8%)	69 (34.5%)
BMI (kg/m^2^), mean ± SD	27.6 ± 5.3	24.0 ± 4.3
HbA1c (%), mean ± SD	7.1 ± 0.9	7.3 ± 4.6
Insulin therapy, n (%)	43 (25.1%)	141 (70.5%)
CPR (ng/mL), mean ± SD	4.2 ± 3.6	3.9 ± 2.4
Duration of type 2 diabetes (years), mean ± SD	9.8 ± 8.0	14.9 ± 9.9
Number of oral hypoglycemic agent, mean ± SD	2.8 ± 1.5	2.5 ± 1.4
**Smoking**		
Smoking history, (%)	112 (65.5%)	124 (62.0%)
Pack years, mean ± SD	30.2 ± 23.3	21.7 ± 23.5
**Lung Function**		
COPD, n (%)	9 (5.3%)	45 (22.5%)
FEV1/FVC ratio, mean ± SD	81.3 ± 7.2	75.1 ± 10.5
FEV1% predicted (%), mean ± SD	87.3 ± 15.3	90 ± 18.7

COPD: chronic obstructive pulmonary disease, CPR: C-peptide immunoreactivity, FEV1: forced expiratory volume in one second, FVC: forced vital capacity, FEV1% predicted: percent predicted forced expiratory volume in one second, OHA: oral hypoglycemic agent, pack-years = (number of cigarettes smoked per day/20) × number of years smoked.

### Factors influencing respiratory function

There were no significant variables affecting COPD in the non-elderly group (Table 2). In the elderly group, male sex, low BMI, insulin therapy, and increased C-peptide immunoreactivity (CPR) levels were significant variables for COPD. In the non-elderly group, pack-years was a significant variable for reduced FEV1/FVC ratio (Table 3). In the elderly group, low BMI, insulin therapy, and increased CPR levels were factors related to reduced FEV1/FVC ratio. The relationship between BMI and FEV1/FVC ratio were evaluated by sex. Regression equations for BMI and FEV1/FVC ratio were as follows; male non-elderly group: FEV1/FVC ratio = 0.17 × BMI + 75.9, male elderly group: FEV1/FVC ratio = 0.42 × BMI + 64.2, female non-elderly group: FEV1/FVC ratio = −0.02 × BMI + 84.1, female elderly group: FEV1/FVC ratio = 0.31 × BMI + 69.4. The effect of BMI on FEV1/FVC ratio was evaluated separately for males and females via an analysis of covariance (ANCOVA) with age group as a covariate. The model incorporated BMI and age group interactions. The results showed that low BMI was a significant factor influencing FEV1/FVC ratio in men (Table 4). Considering the regression equations for BMI and FEV1/FVC ratio, low BMI was a factor influencing reduced FEV1/FVC ratio in men. Neither BMI nor age group was a significant factor for reduced FEV1/FVC ratio in women. In the non-elderly group, age, female sex, and high BMI were found to be factors related to reduced percent predicted forced expiratory volume in one second (FEV1% predicted) (Table 5). In the elderly group, female sex, insulin therapy, increased CPR levels, and pack-years were factors related to reduced FEV1% predicted. The above factors related for COPD and reduced FEV1/FVC ratio and FEV1% predicted by age group are shown in Table 6.

**Table 2:** Factors influencing COPD.

Variables	Exp (B)	p	95% CI
**Non-elderly group**			
Age (years)	1.131	0.203	[0.936–1.367]
Male sex	8.6 × 10^7^	0.998	[0.00-—]
BMI (kg/m^2^)	0.795	0.069	[0.620–1.018]
HbA1c (%)	1.489	0.314	[0.686–3.231]
Duration of diabetes (years)	1.010	0.879	[0.894–1.140]
Insulin therapy	0.000	0.998	[0.00-—]
CPR (ng/mL)	0.748	0.334	[0.415–1.347]
Number of OHA	0.860	0.683	[0.418–1.770]
Pack years	0.973	0.327	[0.921–1.028]
**Elderly group**			
Age (years)	0.922	0.071	[0.845–1.007]
Male sex	2.539	0.041	[1.039–6.206]
BMI (kg/m^2^)	0.825	0.001	[0.738–0.922]
HbA1c (%)	0.835	0.527	[0.478–1.459]
Duration of diabetes (years)	0.987	0.563	[0.944–1.032]
Insulin therapy	3.328	0.01	[1.330–8.330]
CPR (ng/mL)	1.204	0.02	[1.029–1.409]
Number of OHA	0.915	0.553	[0.584–1.226]
Pack years	1.010	0.293	[0.992–1.028]

CPR: C-peptide immunoreactivity, OHA: oral hypoglycemic agent, pack-years = (number of cigarettes smoked per day/20) × number of years smoked.

**Table 3:** Factors influencing FEV1/FVC ratio.

Variables	β	p
**Non-elderly group**		
Age (years)	−0.128	0.166
Male sex	1.792	0.207
BMI (kg/m^2^)	0.037	0.753
HbA1c (%)	−0.433	0.515
Duration of diabetes	0.036	0.648
Insulin therapy	3.523	0.056
CPR (ng/mL)	−0.176	0.250
Number of OHA	0.517	0.215
Pack years	−0.050	0.025
**Elderly group**		
Age (years)	0.283	0.074
Male sex	2.733	0.084
BMI (kg/m^2^)	0.679	<0.001
HbA1c (%)	0.238	0.139
Duration of diabetes	0.039	0.643
Insulin therapy	−5.356	0.003
CPR (ng/mL)	−0.650	0.043
Number of OHA	0.019	0.974
Pack years	−0.032	0.374

CPR: C-peptide immunoreactivity, FEV1: forced expiratory volume in one second, FVC: forced vital capacity, OHA: oral hypoglycemic agent, pack-years = (number of cigarettes smoked per day/20) × number of years smoked.

**Table 4:** Influence of BMI and age on FEV1/FVC ratio by sex.

Variables	df	F value	p
**Male**			
BMI (kg/m^2^)	1	5.968	0.015
Age class (Elderly/Non-elderly)	1	3.432	0.065
BMI x Age class*	1	1.071	0.302
**Female**			
BMI (kg/m^2^)	1	0.984	0.324
Age class (Elderly/Non-elderly)	1	3.724	0.056
BMI x age class*	1	1.237	0.262

*Interaction between BMI and age class, FEV1: forced expiratory volume in one second, FVC: forced vital capacity.

**Table 5:** Factors influencing FEV1% predicted.

Variables	β	p
**Non-elderly group**		
Age (years)	−0.497	0.004
Male sex	10.868	<0.001
BMI (kg/m^2^)	−0.476	0.024
HbA1c (%)	−2.447	0.058
Duration of diabetes	0.135	0.375
Insulin therapy	−4.201	0.300
CPR (ng/mL)	0.198	0.519
Number of OHA	0.225	0.787
Pack years	−0.057	0.241
**Elderly group**		
Age (years)	0.196	0.465
Male sex	9.413	0.002
BMI (kg/m^2^)	0.509	0.090
HbA1c (%)	0.062	0.815
Duration of diabetes	−0.062	0.655
Insulin therapy	−9.236	0.002
CPR (ng/mL)	−1.291	0.015
Number of OHA	0.525	0.581
Pack years	−0.162	0.005

CPR: C-peptide immunoreactivity, FEV1% predicted: percent predicted forced expiratory volume in one second, OHA: oral hypoglycemic agent, pack-years = (number of cigarettes smoked per day/20) × number of years smoked.

**Table 6:** Factors related to reduced respiratory function by age class.

Indicators of respiratory function	Factors relate to reduced respiratory function	
	Non-elderly group	Elderly group
COPD	—	Male sex Low BMI Insulin therapy High CPR
FEV1/FVC ratio	High pack years	Low BMI Insulin therapy High CPR
FEV1% predicted	Age Female sex High BMI	Female sex Insulin therapy High CPR High pack years

CPR: C-peptide immunoreactivity, FEV1: forced expiratory volume in one second, FVC: forced vital capacity, FEV1% predicted: percent predicted forced expiratory volume in one second, Pack-years = (number of cigarettes smoked per day/20) × number of years smoked.

## Discussion

Among the patients with T2DM who underwent respiratory function tests, COPD was found in 5.3% of the non-elderly group and 22.5% of the elderly group. We found that low BMI, insulin therapy, and increased CPR levels were factors related to COPD and reduced FEV1/FVC ratio in the elderly group. High BMI was a factor related to reduced FEV1% predicted in the non-elderly group. Female sex was determined to be a factor related to reduced FEV1% predicted in both age groups.

In the NICE study in Japan, the prevalence of COPD was 8.6% in people over 40 years of age and 17.4% in those over 70 years of age^6^. In our study, the prevalence of COPD in the same age class was found to be 14.6% for those over 40 years of age and 20.9% for those over 70 years of age (22.5% for those over 65 years of age). Thus, our results indicate that the prevalence of COPD in T2DM patients may be higher than in the general population.

This study showed an association between increased CPR levels and decreased respiratory function in the elderly group. CPR levels reflect increases in insulin secretion in response to insulin resistance^19^. Therefore, our results indicate that insulin resistance is a factor related to COPD and reduced FEV1/FVC ratio. T2DM impairs both large and micro-vessels; thus, alveolar capillary beds might also be affected^20,21^. Indeed, diffusing capacity of lung for carbon monoxide (DLCO), an indicator of CO binding from alveoli to pulmonary capillary hemoglobin, was reduced in diabetic patients^22^. DLCO values also vary with morphological or functional changes in alveolar capillaries^23^. In pulmonary tissues of diabetic model animals, inflammatory changes in pulmonary vascular tissues, decreased antioxidant enzyme activity, thickening of pulmonary capillary basement membrane and decreased ventilation capacity in alveoli have been reported^24^. Considering the above, the influence of T2DM on respiratory function may be due to alveolar morphological changes through increased oxidative stress associated with inflammation. Furthermore, it has been reported that phrenic nerve conduction velocity was decreased in diabetic patients^25^. These effects may be related to the relationship between increased CPR and decreased respiratory function found in this study.

We found low BMI to be a factor related to reduced respiratory function in elderly patients with T2DM. Table 7 shows patient distribution and BMI according to severity of COPD. In this study, there were only two patients with stage III and no patient with stage IV. Due to the small sample size of severe cases, no clear result was obtained; however, mean BMI was relatively low in patients with Stage III compared with those of Stage I or II in the elderly group. This may be due to systemic vulnerability associated with aging. The prevalence of frailty in diabetic patients is reportedly three to five times higher than in non-diabetic patients^26,27^. Frailty is caused by various factors including loss of muscle mass. Insulin increases protein synthesis and decreases protein degradation in the muscle; thus, insulin resistance may lead to reduce muscle mass and strength in T2DM^28,29^. Furthermore, insulin-like growth factor-1 (IGF-1), which affects muscle and bone strength, also decreases in T2DM patients^29,30^. Thus, T2DM patients are prone to frailty, which in turn affects respiratory function and physical activity, leading to further complications^31^. Therefore, low BMI as a factor related to reduced respiratory function in elderly patients is considered to reflect systemic vulnerability.

**Table 7:** Patient distribution and BMI according to severity of COPD.

	Total	GOLD stage			
		Stage I	Stage II	Stage III	Stage IV
**Non-elderly group (n = 171)**					
n (%) of patients with COPD	9	1 (11.1%)	8 (88.9%)	0	0
BMI (kg/m^2^), mean ± SD	27.2 ± 6.6	23.1	27.7 ± 6.9		
**Elderly group (n = 200)**					
n (%) of patients with COPD	45	16 (35.6%)	27 (60%)	2 (4.4%)	0
BMI (kg/m^2^), mean ± SD	22.7 ± 3.7	22.2 ± 3.3	23.2 ± 3.9	19.6 ± 0.8	

GOLD: Global Initiative for Chronic Obstructive Lung Disease, Stage I: FEV1% predicted ≥ 80%, Stage II: 50% ≤ FEV1% predicted < 80%, Stage III: 30% ≤ FEV1% predicted < 50%, Stage IV: FEV1% predicted < 30%.

Contrastingly, high BMI was found to be a factor related to reduced FEV1% predicted in the non-elderly group. Fat accumulation in the rib cage and visceral cavity reduces respiratory function by adversely affecting functional residual capacity^14,32^. While the mean BMI for the general Japanese population is 23.7 kg/m^2^ for men and 22.5 kg/m^2^ for women^33^, the non-elderly group in this study had a mean BMI of 27.4 kg/m^2^. Thus, high BMI might have affected reduced respiratory function in the non-elderly group. However, this relationship was not found for FEV1/FVC ratio. Since increased body fat decreases FVC and FEV1^31,32^, it is possible that sensitivity for respiratory function was higher for FEV1% predicted compared to FEV1/FVC ratio in the study population. Low BMI was a factor related to reduced FEV1/FVC ratio in men of all ages. This indicates that the influence of low BMI was a dominant for reduced FEV1/FVC ratio in men in the integrated evaluation of high BMI in the non-elderly group and low BMI in the elderly group, both of which were factors related to reduced FEV1/FVC ratio. Therefore, although low BMI was a factor related to reduced FEV1/FVC ratio in men, it should be noted that the effect of BMI differs between non-elderly and elderly patients.

Male sex was a factor related to COPD only in the elderly group and female sex was a factor related to reduced FEV1% predicted in both age groups. Smoking rates in Japan were higher for men (27.8%) than for women (8.7%) in 2018^34^ and correspondingly, there are more men with COPD (183,000) than women (79,000)^35^. However, women are more sensitive to cigarette smoke than men, and thus, they likely to be more susceptible to respiratory function problems^36^. Taking these data into account, our results suggest that men are at higher risk of developing COPD, but women are at higher risk of reduced respiratory function, including non-COPD.

In the analysis of all patients, we found a weak positive correlation between the FEV1/FVC ratio and BMI, and no significant correlation between FEV1% predicted and BMI (data not shown). However, the effect of BMI on respiratory function differs between elderly and non-elderly patients, and also varies by sex. These results indicate that, in T2DM patients, factors affecting respiratory function differ depending on their background, making it difficult to elucidate the relationship between T2DM and respiratory function. An important finding in this study is that the factors affecting respiratory function in T2DM patients differed by age and sex.

This was a single-site study with a small sample size of 371. The mean BMI for patients was 27.4 for the non-elderly group and 24.0 for the elderly group; the analysed population seems more likely to be obese compared with the general population. Therefore, the results of our study need to be interpreted with consideration of these limitations.

Both T2DM and COPD are affected by BMI, and each disease affects each other. Therefore, in patients with T2DM, respiratory function tests are important for risk management of COPD as well as T2DM itself. Our study showed that T2DM was a factor related to COPD and that BMI needs to be managed according to patient age and general condition.

## Methods

### Patients

Eligible patients for this study included those with T2DM who had visited Okamoto Internal Medicine Clinic, Shizuoka, Japan, from April 2017 to September 2018 and met the following inclusion criteria: (1) patients with established diagnosis of T2DM ≥ 40 years of age, (2) patients without respiratory disease or those having a history of disease other than COPD that may affect the spirometry, and (3) provision of written consent for a respiratory function test.

This study was conducted in compliance with the Declaration of Helsinki and according to the Ethical Guidelines for Medical and Health Research Involving Human Subjects established by the Ministry of Health, Labour, and Welfare in Japan. The study protocol was approved by the ethical review committees of the Seishinkai Group and Okamoto Internal Medicine Clinic. Written informed consent was obtained from all study patients.

## Methods

Respiratory function tests using spirometry were performed after bronchodilation in eligible patients. FEV1/FVC ratio and FEV1% predicted were measured, and the absence or presence of COPD was ascertained. COPD was diagnosed when the FEV1/FVC ratio after inhalation of bronchodilator was less than 70% and other diseases that might result in obstructive disorders were ruled out. Patient background data, including age, sex, BMI, duration of T2DM, number of oral hypoglycemic agents (OHAs), smoking history, pack-years, presence of insulin therapy, HbA1c and CPR levels, were collected. Current and former smokers were designated as having a smoking history. The number of OHAs was the sum of prescribed agents including metformin, sulfonylureas, thiazolidines, alpha-glucosidase inhibitors, dipeptidyl peptidase-4 inhibitors, sodium-glucose cotransporter-2 inhibitors, and glinides. The analysis was carried out in the patient population with data for all items above. The analytical population was divided into the non-elderly and the elderly groups. The factors influencing COPD, FEV1/FVC ratio, and FEV1% predicted were evaluated for each age group. The influence of age and BMI on FEV1/FVC ratio was assessed by sex. FEV1% predicted was calculated as percentage of measured value of FEV1 to FEV1 predicted value. FEV1% predicted was calculated as the percentage of the measured value of FEV1 of the FEV1 predicted value. The FEV1 predicted value was estimated from the following formulas by The Japanese Respiratory Society^37^: Male: FEV1 (L) = 0.036 × height (cm) − 0.028 × age − 1.178, Female: FEV1 (L) = 0.022 × height (cm) − 0.022 × age − 0.005. Pack-years were calculated using the following formula: pack-years = (number of cigarettes smoked per day/20) × number of years smoked.

### Statistics

Descriptive statistics were expressed as n (%) and mean ± standard deviation. Factors influencing COPD were evaluated using stepwise multiple logistic analysis with age, sex, BMI, HbA1c, duration of T2DM, insulin therapy, number of OHAs, CPR level, and pack-years as independent variables. For the evaluation of factors influencing FEV1/FVC ratio and FEV1% predicted, stepwise multiple regression analysis was used with the same variables as the independent variables. An ANCOVA with age as a covariate was used to evaluate the effect of BMI on FEV1/FVC ratio. Two-sided α was set to be 0.05. Statistical analysis was performed using SPSS Statistics ver. 25 (IBM Corp., Armonk, N.Y., USA).

## Data Availability

The datasets analyzed will be available from the corresponding author on reasonable request.
