# Publisher Correction: Characteristics of factors for decreased lung function in elderly patients with type 2 diabetes

**DOI:** 10.1038/s41598-020-59782-x

**Published:** 2020-02-13

**Authors:** Masaki Ishii, Yasuhiro Yamaguchi, Hironobu Hamaya, Sumito Ogawa, Mitsuo Imura, Masahiro Akishita

**Affiliations:** 1 The Department of Geriatric Medicine, The University of Tokyo, Tokyo, Japan; 2 Division of Department of Respiratory Medicine, Jichi Medical University Saitama Medical Center, Saitama, Japan; 3 Okamoto Internal Medicine Clinic, Shizuoka, Japan

Correction to: *Scientific Reports* 10.1038/s41598-019-56759-3, published online 27 December 2019

The original version of this Article contained a typographical error in the spelling of the author Masahiro Akishita, which was incorrectly given as Akishita Masahiro. This has now been corrected in the PDF and HTML versions of the Article.

